# Neighborhood‐Level Variation in the Effectiveness of Intensive Health Behavior and Lifestyle Treatment for Pediatric Obesity

**DOI:** 10.1002/oby.70292

**Published:** 2026-09-17

**Authors:** Grace Wang, Stephanie Samuels, Kaitlin R. Maciejewski, Pamela Hu, Mary Savoye, Mona Sharifi

**Affiliations:** ^1^ Yale School of Medicine New Haven Connecticut USA; ^2^ Section of Pediatric Endocrinology & Diabetes, Department of Pediatrics Yale School of Medicine New Haven Connecticut USA; ^3^ Yale Center for Analytical Sciences Yale School of Public Health New Haven Connecticut USA; ^4^ Section of General Pediatrics, Department of Pediatrics Yale School of Medicine New Haven Connecticut USA

**Keywords:** behavioral intervention, geocoding, pediatric obesity

## Abstract

**Objective:**

This study assessed variation in BMI change by neighborhood characteristics of participants in Bright Bodies, a family‐based intensive health behavior and lifestyle treatment for pediatric obesity.

**Methods:**

For first‐time participants (2008–2018) with initial BMI ≥ 85th percentile, we geocoded addresses and assigned Child Opportunity Index (COI) and COI‐Healthy Environment (COI‐HE) scores (Low/Moderate/High). Using multivariable mixed effect linear regression, we assessed monthly change in BMI percent of the 95th percentile (%BMIp95) and extended BMI *z* score by COI and COI‐HE.

**Results:**

Of 391 youth (mean ± SD age 11.7 ± 2.8 years, 60.1% female), 80.8% resided in Low COI and 66.5% in Low COI‐HE neighborhoods. Monthly %BMIp95 change differed by COI (*p* = 0.039 for interaction) and COI‐HE (*p* = 0.004). Mean %BMIp95 decreased in all subgroups; greatest reductions were in High COI (−1.86 [95% CI: −2.40, −1.33]) and High COI‐HE groups (−2.32 [−2.78, −1.85]) and lowest in Moderate COI (−1.17 [−1.59, −0.75]) and Low COI‐HE groups (−1.53 [−1.75, −1.32]). Change in extended BMI *z* score differed significantly between COI‐HE subgroups only (*p* < 0.001).

**Conclusions:**

Bright Bodies participation was associated with BMI reduction among all COI/COI‐HE subgroups, but the highest COI/COI‐HE groups had the greatest reductions. Modifications to intensive health behavior and lifestyle treatments should address inequities in access to built environments that facilitate healthy, active living.

## Study Importance

- What is already known?
  ○ Bright Bodies is a high‐intensity, multicomponent, intensive health behavior and lifestyle treatment program proven effective in the context of real‐world adaptations.
  ○ Social determinants of health, such as neighborhood characteristics and built environment, are strongly associated with pediatric obesity and can modify the effectiveness of treatment.
- What does this study add?
  ○ In this real‐world observational study of 391 children enrolled in Bright Bodies sessions between 2008 and 2018, we observed improvements in BMI measures among participants across all levels of neighborhood opportunity and neighborhood environment quality.
  ○ We observed significant differences in BMI change by overall neighborhood opportunity and neighborhood environment quality, with the greatest reductions in BMI among those from the neighborhoods with highest opportunity.
- How might these results change the direction of research or the focus of clinical practice?
  ○ Our findings suggest that access to healthy food, green spaces, and walkable built environments may modify program effectiveness.
  ○ Future modifications to Bright Bodies and similar pediatric lifestyle interventions should seek to address inequities in participants' access to built environments that facilitate healthy, active living.

## 1 Introduction

The prevalence of pediatric obesity has escalated in recent decades to the levels of a national epidemic, such that obesity and overweight now affect more than one‐third of United States children aged 2–19 years [1]. The prevalence of overweight and obesity is disproportionately higher among Hispanic and non‐Hispanic Black youth and children in lower‐income households [2, 3]. While historically obesity has been stigmatized as a reversible consequence of individual choices, robust evidence supports the complex environmental, socioeconomic, physiologic, and genetic facets of one's predisposition, including the pervasive structural inequities that underlie alarming and persistent racial, ethnic, and socioeconomic disparities in both obesity and its myriad of serious short‐ and long‐term health consequences [3].

The 2023 American Academy of Pediatrics guidelines on the management of pediatric obesity and a 2024 US Preventive Services Task Force report both endorse the value of intensive health behavior and lifestyle treatments (IHBLTs) [4, 5]. Yet access to and availability of IHBLTs remain limited, particularly for children who live in neighborhoods disproportionately impacted by obesity [6, 7].

One well‐established IHBLT is Bright Bodies, a family‐based group program developed at Yale in the 2000s. In a randomized controlled trial (RCT) published in 2007, Bright Bodies, compared with biannual visits in a multidisciplinary obesity clinic, improved body mass index (BMI), percent body fat, and insulin resistance for individuals ages 8–16 years old with obesity from diverse racial and ethnic backgrounds [8], with maintenance of effects 1 year after active intervention [9]. In a recent follow‐up study, we observed sustained effectiveness in a real‐world setting for participants across 11 years since the initial RCT [10]. However, no study has examined the neighborhood characteristics of Bright Bodies participants or the extent to which those characteristics may impact program effectiveness.

Geographic Information System (GIS) analyses have been used to visualize the spatial distribution and density of obesity and to demonstrate that neighborhood characteristics such as walkability, park access, and proximity to supermarkets are associated with higher obesity prevalence and greater response to interventions [11, 12, 13, 14, 15, 16, 17]. This knowledge can help inform equitable allocation of resources for specific, complex, real‐world populations [18, 19]. In the geospatial study of pediatric obesity, existing literature predominantly examines the impact of individual neighborhood characteristics, rather than considering the combined effect of multiple factors, which are likely cumulative [19]. One robust dataset that is a multidimensional composite index of neighborhood features (“indicators”) that impact child health is the Child Opportunity Index (COI), which reflects the compounding benefits that multiple sources of advantage provide [20]. The COI incorporates 29 neighborhood‐level indicators of health categorized into three domains: education, health and environment, and social and economic context. Data from which the COI is constructed are drawn from public sources, and components are weighted as a function of correlation between each indicator and various socioeconomic and health outcomes based on prior literature. Prior studies have shown that residence in higher‐COI neighborhoods is associated with lower baseline BMI and healthier BMI trajectories among children and adolescents [21, 22].

In this study, our primary aim was to examine neighborhood‐level variation in the real‐world effectiveness of past in‐person Bright Bodies programs (2008–2018), defined as within‐participant changes in BMI over time, considering specifically the overall COI, as well as the COI‐Healthy Environment (COI‐HE), a subdomain reflecting food quality, extreme heat exposure, nature access, and walkability. We hypothesized that the effectiveness of Bright Bodies would be modified by neighborhood COI and COI‐HE levels, reflecting the association of environmental and socioeconomic factors with the pathogenesis and clinical course of pediatric obesity.

## 2 Methods

### 2.1 Participants

We included all first‐time Bright Bodies participants who (1) enrolled and attended ≥ 1 orientation, nutrition education, or physical activity class between 2008 and 2018; and (2) had an initial BMI measurement ≥ 85th percentile for age/sex. We excluded participants (1) for whom first‐program attendance data were unavailable or (2) who were concurrently enrolled in an RCT assessing the efficacy of Bright Bodies for pediatric patients with early signs of impaired glucose tolerance [23]. One participant with an address outside the state of Connecticut (CT) was excluded from the study, as the state‐normed COI data were deemed most appropriate for our analysis of predominantly CT residents. Historical address data from within 1 year of Bright Bodies enrollment were extracted from patients' electronic health records or Bright Bodies program records. Of all 396 eligible participants, we successfully extracted address data for 391 (98.7%) participants, who were included in our final sample for analysis.

The Yale University Institutional Review Board (IRB) reviewed and approved the original Bright Bodies Data Review study (#2000025507). A waiver of consent was granted.

### 2.2 Bright Bodies Intervention

The intervention is described in more detail in prior publications [8, 10]. Bright Bodies programs during the years 2008–2018 ranged from 6 to 12 consecutive weeks due to constraints around holidays and weather.

### 2.3 Data Collection and Measures

Demographic, anthropometric, and participation data were routinely collected during each Bright Bodies program. The date of the first available weight measurement for each participant was interpreted as the date of enrollment, as late enrollment in programs was allowed. BMI categorical classifications were determined using the percentiles described in Ryder et al. (Overweight, Class 1 Obesity, Class 2 Obesity, Class 3 Obesity) [24]. Enrollment year was categorized as early (2008–2010), middle (2011–2014), or late (2015–2018).

Weight was measured at enrollment and on the final day of the program using the TBF 300 analyzer (Tanita, Arlington Heights, IL) [8]. Height was measured using a Harpenden stadiometer (Cambridge, MD). These instruments were also used for the original 2007 Bright Bodies RCT [8]. The primary outcome was the monthly change in BMI during the participants' first Bright Bodies program, examined as a percent of the 95th percentile (%BMIp95), as well as the extended BMI *z* score (extBMIz) [25, 26]. %BMIp95 was examined because existing literature provides guidance for interpretations of clinical meaningful change [27]. We also examined extBMIz because it is derived from updated Centers for Disease Control and Prevention extended growth charts based on newer data for children and adolescents with obesity and addresses limitations of prior BMI *z* scores in assessing change in adiposity over time in children with severe obesity [28, 29].

### 2.4 Geocoding

Participants' residential addresses were reviewed for completeness and geocoded using ArcGIS Pro 3.0 (Esri Inc., Redlands, CA). We then linked geocoded addresses to 2020 US census tracts [30]. For each geocoded address, an Origin–Destination Cost Matrix was used to calculate the one‐way driving time to the in‐person Bright Bodies location, the Celantano School in New Haven, CT.

Next, we determined the COI categorical score for the geocoded census tract corresponding to each participant's address. Details of how the COI composite index is constructed are available publicly via DiversityDataKids (for index domains, see Table S1) [20]. The COI scores were year‐matched to year of participation as closely as possible. Participants who enrolled in 2008–2012 were matched to 2012 COI observations, as that is the earliest date of data in the COI 3.0. The COI‐level distributions of participants assigned scores based on 2012 observations, versus those assigned based on year‐matched observations, are shown in Table S2. We collapsed the “Very High” COI category with “High” to address small sample sizes, and we also collapsed the “Very Low” category with “Low” to preserve the original distribution of categories. Existing literature has used this redistribution of categories [31].

We additionally examined the Heathy Environment subdomain indicator of the COI (COI‐HE) due to its direct relevance to the Bright Bodies intervention, which is centered around regular exercise, healthy eating with a nondiet approach, and behavioral modification. This indicator accounts for the fast‐food restaurant density, healthy food retailer density, extreme heat exposure, “NatureScore,” and the walkability of each census tract (Table S3). The methods associated with assigning COI‐HE for each participant were analogous to those for COI described earlier. High COI and High COI‐HE scores indicate higher neighborhood opportunities and access to resources.

### 2.5 Statistical Analyses

Baseline characteristics of participants were summarized using means and standard deviations (SD) for continuous variables and frequencies and proportions for categorical variables. Driving time to the in‐person Bright Bodies location was summarized using medians (interquartile range [IQR]) to reduce the influence of outliers. We used linear mixed effects models to examine monthly change in %BMIp95 during participants' first Bright Bodies program. Separate models were created for COI and COI‐HE, each adjusting for age, sex, season, year of first session, starting %BMIp95, race/ethnicity, and insurance type. While some existing literature has demonstrated collinearity between race/ethnicity and socioeconomic status and the overall COI [32, 33, 34], we did not observe any significant differences in our results when running our model without controlling for race/ethnicity and insurance status. Models were replicated to examine monthly change in extBMIz, adjusting for the same covariates, with extBMIz [35] included in place of starting %BMIp95.

All statistical analyses were performed using SAS software version 9.4 (SAS Institute Inc., Cary, NC). The two‐sided alpha level was set as ≤ 0.05 to determine statistical significance.

## 3 Results

### 3.1 Sample Characteristics

**FIGURE 1:**
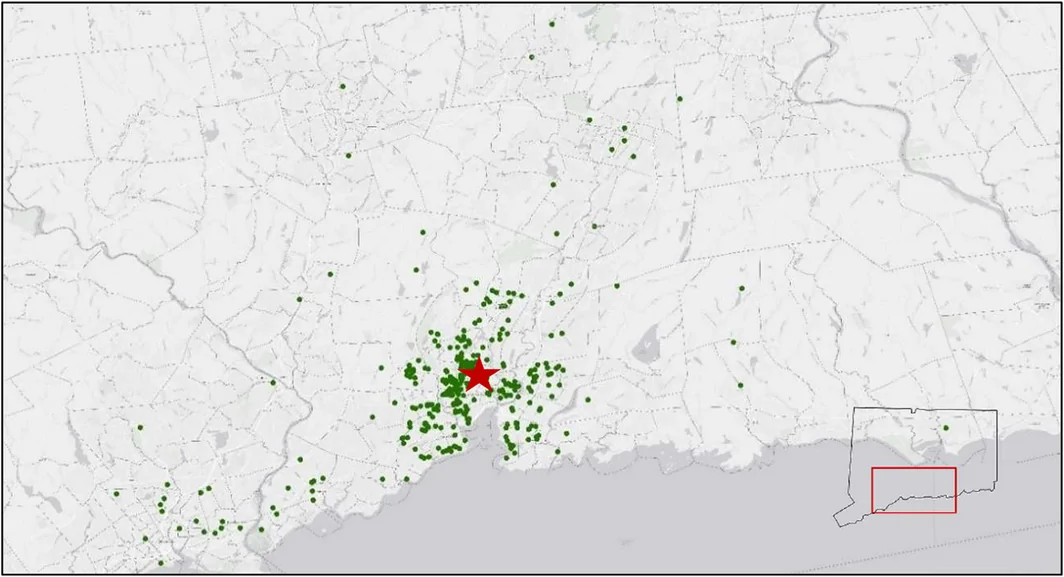
Geographic distribution of Bright Bodies participants, 2008–2018. Key: 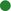 Geocoded addresses of participants (*N* = 391, outliers not shown for image quality); 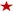 Celantano School, New Haven, CT; 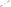 2020 census tract lines. [Color figure can be viewed at wileyonlinelibrary.com]

The sample included 391 youth, with a mean ± SD age 11.7 ± 2.8 years (Table 1). Most participants (60.1%) identified as female, 53.7% had public insurance, 50.4% had severe (Class 2 or 3) obesity, 37.3% were non‐Hispanic Black, and 25.8% were Hispanic/Latino. The median (IQR) one‐way driving time for participants was 11.6 min (7.6–15.0 min). Participants lived in 108 unique census tracts, and the geographic distribution of participants is shown visually in Figure 1. Most participants (80.8%) resided in neighborhoods categorized as Low COI (low opportunity), 10.5% in Moderate, and 9.7% in High (Table 1). The classification of participants' neighborhoods by COI‐HE category was 66.5% Low, 22.5% Moderate, and 11.0% High. Participants had a mean of 5 (SD 2.8) BMI data points during the 13‐week post‐enrollment period.

**TABLE 1:** Characteristics of new Bright Bodies participants, 2008–2018, *N* = 391.

Participant characteristics	No. (%) or mean (SD)
Age, years	11.7 (2.8)
Female sex	235 (60.1%)
Race and ethnicity	
Non‐Hispanic White	89 (22.8%)
Non‐Hispanic Black	146 (37.3%)
Hispanic or Latino	101 (25.8%)
Other/unknown	55 (14.1%)
Health insurance type	
Public	210 (53.7%)
Private	134 (34.3%)
Other	47 (12.0%)
Baseline anthropometric measures	
%BMIp95	142.9 (27.4)
BMI *z* score	2.5 (0.4)
Baseline BMI category	
Overweight	18 (4.6%)
Class 1 obesity	60 (15.4%)
Class 2 obesity	116 (29.7%)
Class 3 obesity	197 (50.4%)
Year of first program	
Early (2008–2010)	136 (34.8%)
Middle (2011–2014)	110 (28.1%)
Late (2015–2018)	145 (37.1%)
Season of enrollment	
Fall	150 (38.4%)
Spring	85 (21.7%)
Winter	156 (39.9%)
Child Opportunity Index	
Low	316 (80.8%)
Moderate	41 (10.5%)
High	34 (8.70%)
Child Opportunity Index‐Healthy Environment	
Low	260 (66.5%)
Moderate	88 (22.5%)
High	43 (11.0%)
One‐way driving time, min^a^	
Median (IQR)	11.6 (7.6–15.0)

Abbreviation: %BMIp95, BMI percent of the 95th percentile.

^a^ To the in‐person Bright Bodies location, the Celentano School, New Haven, CT.

### 3.2 Neighborhood‐Level Variation in Effectiveness of Bright Bodies

#### 3.2.1 *%* BMIp95

**FIGURE 2:**
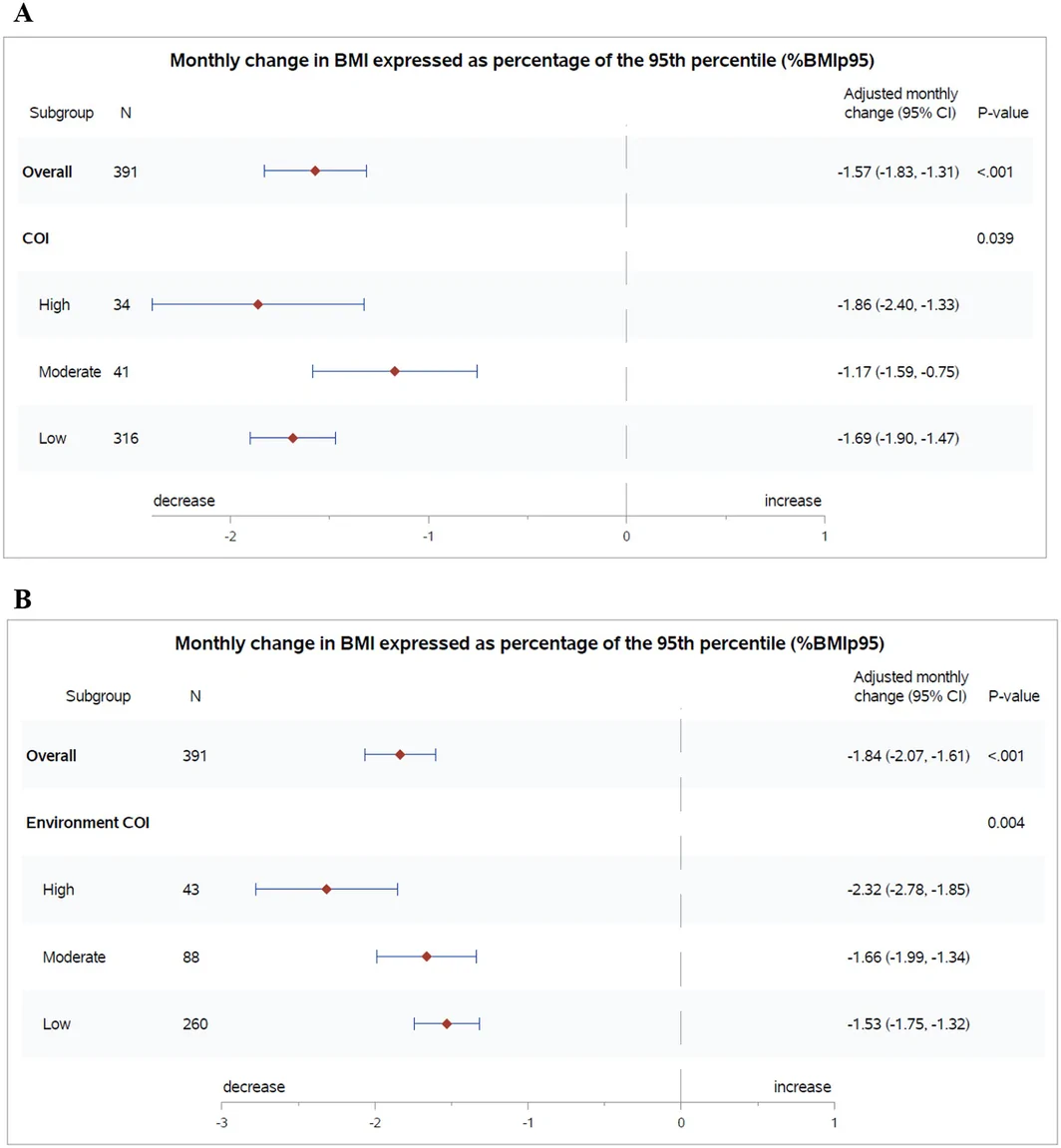
Participants' adjusted monthly change in BMI percent of the 95th percentile (%BMIp95) by neighborhood Child Opportunity Index (COI) and COI‐Healthy Environment (COI‐HE). (A) COI results. (B) COI‐HE results. Adjusted for age, sex, season and year of enrollment, starting %BMIp95, race/ethnicity, and insurance type. [Color figure can be viewed at wileyonlinelibrary.com]

In the COI model, participants' %BMIp95 reduced on average by 1.57 [95% confidence interval: 1.31, 1.83] per month (Figure 2A). Monthly %BMIp95 change differed by COI (*p* = 0.039 for interaction of COI category and time). In stratified analyses, %BMIp95 decreased in all COI subgroups. Participants from High COI neighborhoods (−1.86 [−2.40, −1.33]) had the greatest magnitude of monthly %BMIp95 reduction, followed by Low COI (−1.69 [−1.90, −1.47]) and Moderate COI (−1.17 [−1.59, −0.75]) subgroups. In post hoc analyses, we found no statistically significant difference in change in %BMIp95 among Low and Very Low COI groups (pairwise *t*‐test −0.05 [−0.38, 0.28]; *p* = 0.77).

In the COI‐HE model, participants' %BMIp95 reduced on average by 1.84 [1.61, 2.07] per month (Figure 2B). Monthly %BMIp95 change differed by COI‐HE (*p* = 0.004 for interaction). In stratified analyses, %BMIp95 decreased in all COI‐HE subgroups. Greatest reductions were observed among those from High COI‐HE neighborhoods (−2.32 [−2.78, −1.85]), followed by Moderate COI‐HE (−1.66 [−1.99, −1.34]) and Low COI‐HE (−1.53 [−1.75, −1.32]) subgroups.

#### 3.2.2 extBMIz

**FIGURE 3:**
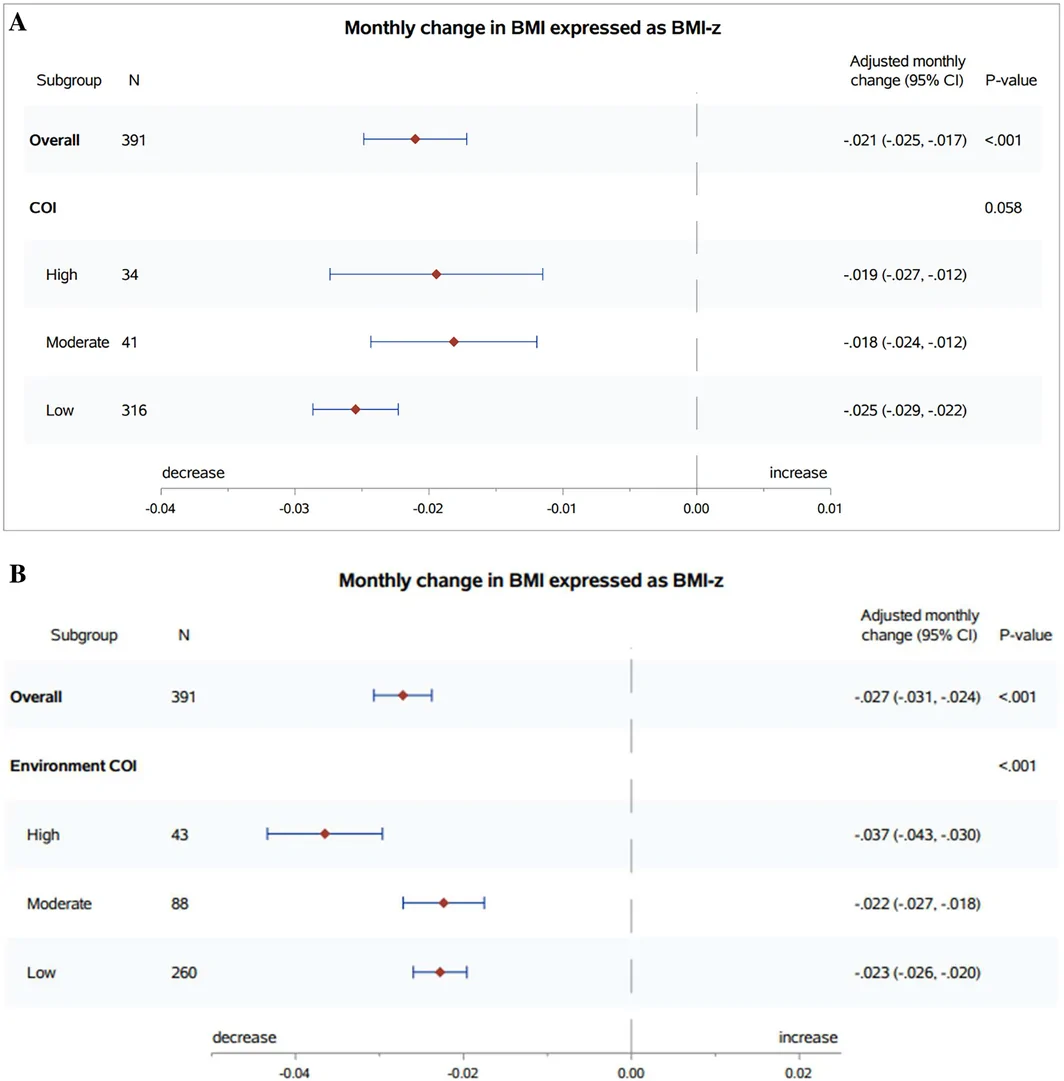
Participants' adjusted monthly change in extended BMI *z* score (extBMIz) by neighborhood Child Opportunity Index (COI) and COI‐Healthy Environment (COI‐HE). (A) COI results. (B) COI‐HE results. Adjusted for age, sex, season and year of enrollment, starting extended BMI *z* score, race/ethnicity, and insurance type. [Color figure can be viewed at wileyonlinelibrary.com]

In the COI model, participants' extBMIz reduced on average by 0.021 [0.017, 0.025] per month (Figure 3A). Monthly extBMIz change was not observed to differ by COI (*p* = 0.058 for interaction). In stratified analyses, extBMIz decreased in all COI groups.

In the COI‐HE model, participants' extBMIz reduced on average by 0.027 [0.031, 0.024] per month (Figure 3B). Monthly extBMI change differed by COI‐HE (*p* < 0.001 for interaction). Greatest reductions were observed among those from High COI‐HE neighborhoods (−0.037 [−0.043, −0.030]), followed by Moderate COI‐HE (−0.022 [−0.027, −0.018]) and Low COI‐HE (−0.023 [−0.026, −0.020]).

## 4 Discussion

In this study of 391 participants in Bright Bodies from 2008 to 2018, we found that the majority of participants resided in areas with low neighborhood opportunity. We observed significant reductions in both measures of BMI among all subgroups of COI and COI‐HE. Change in %BMIp95 differed by COI and COI‐HE subgroup, with the greatest reductions observed among those in High COI and COI‐HE groups. Significant differences in change in extBMIz across subgroups were observed only for COI‐HE, with the greatest reduction among participants in the High COI‐HE subgroup. The demographic characteristics of our study sample are consistent with those of the population which the Bright Bodies program was originally designed to serve, namely the predominantly low‐income, non‐Hispanic Black and Hispanic/Latino youth who are disproportionately affected by obesity in New Haven. Our study highlights that over a decade since the publication of the original Bright Bodies RCT, the program continued to reach the intended population of youth, and participation was associated with BMI reduction, even among youth residing in the neighborhoods with the most limited access to socioeconomic opportunities and healthy environmental resources, as defined by the COI and COI‐HE.

Although it is well established that children residing in under‐resourced communities have greater rates of obesity [2, 3, 21, 22], this study uniquely contributes to the understanding of the role of neighborhood‐level drivers of health within a proven‐effective IHBLT program delivered in a real‐world context over a decade. Our finding that BMI change among participants of Bright Bodies, using two measures of BMI, differed by COI‐HE is likely attributable to the role that local food and built environments play in not only the pathogenesis and progression of pediatric obesity, but also in the practical ability of youth participating in IHBLTs to implement health behavioral changes that lead to sustainable weight change. While there is a substantial body of literature supporting the association between healthy neighborhood environments and children's weight status [12, 13, 15], few studies include longitudinal measures that specifically examine the influence of neighborhood‐level factors on the effectiveness of treatment for overweight and obesity. However, our results are consistent with the limited prior results. For example, one prospective study explored the relationship between weight status of children and changes in neighborhood characteristics, finding some association between healthy weight trajectory and healthy food access, and a more robust association with walkability [14]. Another study demonstrated that among participants enrolled in an obesity management study, those with higher proximity to supermarkets had greater improvements in both fruit and vegetable intake and weight status [17]. A qualitative study, in which both children and their parents were interviewed, supports our hypotheses that many barriers to behavior change crucial for the effectiveness of lifestyle programs for pediatric obesity are rooted in neighborhood characteristics such as the availability of safe, low‐cost outdoor physical activities [36].

Although we observed similar, statistically significant interactions of COI‐HE and change in both %BMIp95 and extBMIz, we only observed a statistically significant interaction of COI and the %BMIp95 outcome but not extBMIz. This may be explained by the differences in statistical derivation of these metrics. %BMIp95 has no upper limit and scales linearly with BMI. In contrast, extBMIz exhibits some compression at extremely high BMI, though less so than standard BMI *z* scores. This may have led to a type II error in our COI results, where the *p* value for interaction was larger than for the COI‐HE results and more prone to bias with our small sample size.

Our finding that participants in Moderate COI neighborhoods had a lower magnitude of mean reduction in %BMIp95 than those in Low COI neighborhoods was unexpected. Given this study is an exploratory analysis with small samples of participants from High and Moderate level neighborhoods, we cannot make definitive conclusions about the relationship between COI and the effect of Bright Bodies. Census tract‐level COI assignments are subject to ecological fallacy and unmeasured confounding as possible sources of bias, where the circumstances of an individual participant in Bright Bodies may differ meaningfully from the average experience of residents in a census tract. Neighborhoods labeled as Moderate COI may contain higher variation in how individuals experience neighborhood characteristics included in the COI (e.g., socioeconomic diversity) than in neighborhoods labeled Low or High. For instance, the Prospect Hill census tract in New Haven, from which our sample included youth, is labeled as Moderate COI. This census tract has a high standard deviation in median income of $27,000 and a relatively high Gini income inequality score of 0.63 compared with the CT average score of 0.42 in 2020 (min 0.28, max 0.76) [37]. In our study with relatively small sample sizes in High and Moderate COI subgroups, this heterogeneity may be an unmeasured source of confounding. Subsections of Moderate COI census tracks may better align with Low COI neighborhoods and others with High COI neighborhoods within a single census track. We suspect that children with obesity are more likely to be in the lower‐income households and lower‐opportunity subsections within these census tracts, yet the lack of individual‐level measures of socioeconomic status and more granular neighborhood information limits our ability to assess these hypotheses. Additionally, with respect to variation between the patterns observed in COI versus COI‐HE results, the distinct subcategories of the COI may be unequally prone to ecological fallacy. While households in a given neighborhood may have substantial differences in socioeconomic status, their experience with the local built environment (e.g., neighborhood walkability) is likely to be much more comparable.

This study's findings should be considered in the context of several limitations in addition to census tract‐level data being subject to ecological fallacy as discussed earlier. First, as previously described [10], this retrospective observational study is limited by data availability, including variation in the number of BMI measurements from each participant, low data availability for long‐term participation in Bright Bodies beyond participants' first program, and the absence of a control group. To address these limitations, we used mixed effects regression models to account for variable intervals between repeated measures of BMI. Although the original Bright Bodies RCT demonstrated weight and BMI increases in the clinical control group, suggesting that the observed improvements in the overall sample were unlikely to be attributable solely to secular trends or regression to the mean, both the original trial and the current study lacked a control group stratified by COI. Therefore, the observed differential BMI improvement across COI strata may reflect unmeasured individual‐ or neighborhood‐level confounders rather than a true differential intervention effect. Future controlled studies with stratification by neighborhood opportunity are needed to confirm whether these differential effects are causally related to the intervention or driven by contextual factors that independently promote healthier weight trajectories. Second, our study includes participants in Bright Bodies programs from 2008 to 2018, while the COI observations are only published for 2012 and onward, which introduces a source of potential misclassification bias. However, we do not expect this to have a major impact on our results given that neighborhoods are not expected to change substantially over increments of < 5 years and the absence of statistically significant difference in the distribution of COI levels among participants in 2008–2012 versus 2013–2018. Third, by acquiring historical address data from within 1 year of Bright Bodies enrollment, we did not account for address changes during the duration of Bright Bodies participation, which likely disproportionately affected those from lower‐COI neighborhoods. However, we would expect moves to be predominantly between neighborhoods of similar COI levels. Finally, our data being from a single site limits the generalizability of our findings. Yet New Haven County, where the original Bright Bodies program is located, is one of the counties in the United States that is most representative of the country as a whole when considering a myriad of economic, generational, racial, religious, ethnic, and political factors [38].

While there are many barriers to healthy living that can only be adequately addressed through changes in policy and city infrastructure, there are possible Bright Bodies program adaptations that may help mitigate the disparities seen in our study between participants from different COI‐HE‐level neighborhoods. For example, the program may evaluate the strategic placement of the in‐person location and choose a local school or other community‐based setting closest to the highest density of regional Low COI‐HE neighborhoods to ease transportation barriers to participation. Second, content delivered in nutrition and behavioral modification classes may be tailored to cover specific food shopping strategies in the specific Low COI‐HE neighborhoods in which the most participants reside. Third, exercise classes may focus on movements that minimize space requirements to help make home exercise a more attractive and sustainable option outside of class participation for participants who lack access to safe recreational spaces outside their homes.

## 5 Conclusion

In conclusion, while previous work has demonstrated ongoing real‐world effectiveness of the Bright Bodies intervention for youth with overweight and obesity, our study highlights the program's reach into the intended populations of youth and neighborhoods disproportionately affected by obesity in the decade of real‐world program delivery following the original randomized trial. Additionally, Bright Bodies participation was associated with mean BMI reduction in all neighborhood COI or COI‐HE subgroups, although those in neighborhoods with greatest opportunity had more substantial decreases in BMI. This study uniquely suggests that IHBLTs like Bright Bodies may enhance effectiveness and equity through proactive consideration of disparities in access to healthy food, green spaces, and walkable built environments.

## Funding

Research reported in this publication was supported by the National Heart, Lung, and Blood Institute of the National Institutes of Health (NHLBI) under Award Number R01HL151603, the American Diabetes Association under Award Number 7‐22‐JDFN‐03, and the Robert Leet and Clara Guthrie Patterson Trust. The NIH and other funders had no role in the design and conduct of this study. The content is solely the responsibility of the authors and does not necessarily represent the official views of the NIH or other funders.

## Conflicts of Interest

Ms. Savoye is the owner of Smart Moves LLC, a company that offers a pediatric weight management program model and educational curriculum. The other authors declared no conflicts of interest.

## Supporting information


**Table S1:** Domains and subcategories of the Child Opportunity Index.
**Table S2:** Child Opportunity Index distributions among first‐time Bright Bodies participants in 2008–2012 and 2013–2018.
**Table S3:** Child Opportunity Index‐Healthy Environment measures.

## Data Availability

The data that support the findings of this study are available from the corresponding author upon reasonable request.
