# Supplementary material for: Neighborhood‐Level Variation in the Effectiveness of Intensive Health Behavior and Lifestyle Treatment for Pediatric Obesity
Source: Obesity (Silver Spring). 2026 Sep 17;34(10):1936–44. doi: 10.1002/oby.70292 (PMC13615428; doi:10.1002/oby.70292)
Supplement: Supplementary file 1 [file OBY-34-1936-s001.docx]

**SUPPLEMENTAL TABLES**

**TABLE S1: Domains and Subcategories of the Child Opportunity Index^a^**

| **Indicator** | **Components** |
| --- | --- |
| EDUCATION |  |
| Early childhood education | % Private pre-K enrollment, % public pre-K enrollment |
| Elementary education | Math and reading standardized test scores (raw, growth, and poverty-adjusted) |
| Secondary, post-secondary education | % + AP course enrollment, % college enrollment in local institutions, % on-time high school graduation |
| Educational resources | % Adults with BA degree or higher, density of non-profits providing enrichment programs for children, % teachers with 3+ years of experience, % children eligible for free or reduced-price lunch |
| HEALTH AND ENVIRONMENT |  |
| Pollution | Microparticle concentration, ozone concentration, index of toxins released by industrial facilities, number of local Superfund sites |
| Healthy environment | Fast food restaurant density, healthy food retailer density, days of extreme heat exposure, NatureScore, EPA Walkability Index |
| Safety | Community safety non-profit density, density vacant housing |
| Health resources | Health service non-profit density, % population with health insurance |
| SOCIAL AND ECONOMIC |  |
| Employment | Employment rate, high-skill employment rate, median earnings of full-time civilian employees |
| Economic resources | Median household income, % individuals under 100% of the federal poverty limit, % households receiving public assistance, food stamps, SNAP |
| Socioeconomic inequality | % Individuals with high school degree, degrees above BA/BS, % households with very high (>125k) and very low (<20k) incomes |
| Housing resources | % Households with high-speed broadband internet, % housing units with >1 occupant per room |
| Social resources | Prevalence of high socioeconomic friend among low socioeconomic individuals, % households headed by single parent, density nonprofits |
| Wealth | % Housing units that are owner-occupied, aggregate home value, capital income, and real estate taxes by number of children |

Abbreviations: AP= advanced placement; BA= Bachelor of Arts; BS= Bachelor of Science; COI= child opportunity index; EPA= environmental protection agency; pre-K= pre-kindergarten; SNAP= supplemental nutrition assistance program

^a^ Details of data source for each of the above index components can be found in the Child Opportunity Index 3.0 Technical Document Appendix 1, pages 54-66.^1^

**Table S2: Child Opportunity Index Distributions Among First-Time Bright Bodies Participants in 2008-2012 and 2013-2018^a^**

| **COI Level** | **2018-2012**  **n (%)** | **2013-2018**  **n (%)** |
| --- | --- | --- |
| Very Low | 95 (50.3) | 107 (52.5) |
| Low | 58 (30.7) | 57 (27.9) |
| Moderate | 21 (11.1) | 20 (9.8) |
| High | 8 (4.2) | 13 (6.4) |
| Very High | 7 (3.7) | 6 (2.9) |
| Total | 189 | 204 |
| **Chi Squared p-value 0.82**  Abbreviation: COI= child opportunity index  ^a^ Because COI observations are only available from 2012 onwards, participants from the years 2008-2012 were assigned COI scores based on their neighborhood’s score in 2012. Participants from the years 2013-2018 were assigned COI scores based on their neighborhood’s score in their participation year. As shown, we did not observe a statistically significant difference in the distribution of participant addresses among COI categories between participants in 2009-2012 assigned 2012 COI measures, vs. participants in 2013-2018 assigned year-matched measures (Chi Squared p-value 0.82). | | |

**Table S3: Child Opportunity Index Healthy Environment Measures^a^**

| **Indicator** | **Definition** |
| --- | --- |
| Fast food restaurant density | Percentage of restaurants that serve fast food, reversed |
| Healthy food retailer density | Percentage of food retailers selling healthy food |
| Extreme heat exposure | Number of summer days with maximum temperature above 90F, reversed |
| NatureScore | Exposure to healthy natural environments using data on green space, tree canopies, parks, and air, noise, and light pollution |
| Walkability | EPA Walkability Index |

Abbreviations: COI= child opportunity index; EPA= environmental protection agency; F= Fahrenheit

^a^ Details of data source for each of the above index components can be found in the Child Opportunity Index 3.0 Technical Document Appendix 1, pages 58-60.^1^

**REFERENCES**

1.	Institute for Equity in Child Opportunity & Healthy Development at Boston University School of Social Work. Data for a diverse and equitable future. Child Opportunity Index 3.0 Technical Documentation, Appendix 1. [www.diversitydatakids.org/research-library/child-opportunity-index/child-opportunity-index-30-2023-census-tract-data](http://www.diversitydatakids.org/research-library/child-opportunity-index/child-opportunity-index-30-2023-census-tract-data). Updated July 24, 2025. Accessed June 1, 2024.
